# Shift schedules and circadian preferences: the association with sleep and mood

**DOI:** 10.3389/fpubh.2024.1283543

**Published:** 2024-04-29

**Authors:** Jihye Ahn, Hyewon Yeo, Somi Lee, Yunjee Hwang, Sehyun Jeon, Seog Ju Kim

**Affiliations:** ^1^Department of Psychiatry, Samsung Medical Center, Sungkyunkwan University College of Medicine, Seoul, Republic of Korea; ^2^Forest Clinic, Seolleung, Republic of Korea; ^3^Department of Brain & Cognitive Engineering, Korea University, Seoul, Republic of Korea

**Keywords:** shift work (MeSH), circadian preference, insomnia, depression, sleepiness

## Abstract

**Object:**

We explored the circadian preferences of non-shift workers (non-SWs) and various types of shift workers (SWs), and the associations of these preferences with sleep and mood.

**Methods:**

In total, 4,561 SWs (2,419 women and 2,142 men aged 37.00 ± 9.80 years) and 2,093 non-SWs (1,094 women and 999 men aged 37.80 ± 9.73 years) completed an online survey. Of all SWs, 2,415 (1,079 women and 1,336 men aged 37.77 ± 9.96 years) reported regularly rotating or fixed schedules (“regular SWs”), and 2,146 (1,340 women and 806 men aged 36.12 ± 9.64 years) had irregular schedules (“irregular SWs”). Of the regular SWs, 2,040 had regularly rotating schedules, 212 had fixed evening schedules, and 163 had fixed night schedules. All participants completed the Morningness-Eveningness Questionnaire (MEQ) exploring circadian preferences, the short form of the Center for Epidemiological Studies-Depression Scale (CES-D) evaluating depression, the Insomnia Severity Index (ISI), and the Epworth Sleepiness Scale (ESS).

**Results:**

Compared to non-SWs, SWs had lower MEQ scores, i.e., more eveningness, after controlling for age, gender, income, occupation, and weekly work hours (*F* = 87.97, *p* < 0.001). Irregular SWs had lower MEQ scores than regular SWs (*F* = 50.89, *p* < 0.001). Among regular SWs, the MEQ scores of fixed evening and fixed night SWs were lower than those of regularly rotating SWs (*F* = 22.42, *p* < 0.001). An association between the MEQ and ESS scores was apparent in non-SWs (r = −0.85, *p* < 0.001) but not in SWs (r = 0.001, *p* = 0.92).

**Conclusion:**

SWs exhibited more eveningness than non-SWs; eveningness was particularly prominent in SWs with irregular or fixed evening/night shifts. Eveningness was associated with sleepiness only in non-SWs, but not in SWs.

## Introduction

Shift work is essential in modern industrialized societies. More than 25% of the global workforce engages in shift work (1). However, shift work compromises health and wellbeing (2). The work schedules of shift workers (SWs) are not aligned with the normal circadian rhythm (3, 4). Circadian rhythms are endogenous rhythms that repeat over a 24 h cycle and are regulated by exposure to light and dark conditions. Adaptation to shift work requires the modification of the circadian preference (the chronotype) (5), i.e., the preferred sleep and activity times; these synchronize biological events with the circadian rhythm (6). The circadian preference modulates the effects of shift work on sleep (7). However, it remains unclear whether an eveningness or morningness preference aids adaptation to shift work. Morningness indicates a preference for going to bed early and waking up early, whereas eveningness indicates a tendency to sleep at later times, often accompanied by difficulty waking up in the morning (8). Previous studies suggested that eveningness might facilitate night shift work more than morningness, and it might also be associated with higher sleep efficiency (9–11). As eveningness has been associated with mood and sleep problems in the general population, the association between circadian preference and sleep/mood may differ between SWs and non-shift workers (non-SWs). However, other studies suggested that eveningness was not necessarily associated with better adaptation to shift work (11, 12). SWs with high morningness working during both the night and day had better sleep quality (11, 13) and less insomnia (11) than eveningness SWs. Therefore, the associations of the circadian preferences of SWs with sleep and mood require attention; both sleep and mood greatly affect shift work adaptation.

In the real world, shift work patterns are diverse and complex. SW sleep and mood vary according to the work schedule (13). A study of 1,253 Hispanic SWs found that those with irregular or nighttime schedules reported later sleep midpoints and greater sleep variability (14). Furthermore, irregular or nighttime SWs exhibited disturbed or delayed circadian rhythms, perhaps reflecting their circadian preferences, especially eveningness (15). However, the cited study had the limitation of including a relatively small number of SWs (*N* = 447). Additionally, it did not compare groups according to the regularity of shift work. It is thus necessary to investigate the circadian preferences of SWs in larger studies that consider various work schedules, including irregular and nighttime shift schedules.

This study aimed to investigate the associations of circadian preferences with sleep and mood in non-SWs and SWs with regular rotating, irregular rotating, and fixed evening/night schedules. We formulated four hypotheses. First, SWs would show more eveningness than non-SWs. Second, irregular shift schedules would be associated with eveningness. Third, night or evening shift schedules would also be associated with eveningness. Finally, the associations of circadian preferences with sleep and mood would differ by shift schedule.

## Methods

### Participants

Participants were recruited via online advertisements and an online survey company (Macromill Embrain Co. Ltd., Seoul, South Korea). Initially, 1,254 participants (32.58 ± 7.93 years of age; 448 men and 806 women, 961 SWs, and 293 non-SWs) were recruited online and via a hospital bulletin board. A consent form and the questionnaires were sent electronically to all eligible participants. As a bias toward young women was apparent, Macromill Embrain recruited 5,400 additional participants (aged 38.33 ± 9.89 years; 2,693 men and 2,707 women, 3,600 SWs, and 1,800 non-SWs) with biases toward men, middle-aged workers, and non-SWs. The company has enrolled >1 million people in various panels, and invitations were sent by e-mail or text messages to members of panels who had agreed to receive such communications. Those who had not agreed to personal contact could apply via a website. The inclusion criteria were an age of ≥18 years and employment in a full- or part-time job. The exclusion criterion was an inability to complete the online questionnaires. Eleven participants whose work schedules were difficult to classify were excluded. Finally, data for 6,654 participants (aged 37.80 ± 9.73 years; 3,413 women and 3,141 men; 4,561 SWs and 2,093 non-SWs) were analyzed. All procedures adhered to the ethical standards of national and institutional committees on human experimentation and the Declaration of Helsinki 1964 (as revised in 2013). The study protocol was approved by the Institutional Review Board of Samsung Medical Center (Approval no. 2019-04-095), and all participants provided written informed consent.

### Shift work patterns

As the effects of SW circadian preferences would be expected to vary by both work time (night or evening) and work regularity, SW patterns were operationally defined as follows: Individuals who worked only during the daytime (7 a.m.–6 p.m.) were classified as non-SWs (*n* = 2,093; 1,094 women and 999 men aged 37.80 ± 9.73 years). SWs (*n* = 4,561; 2,419 women and 2,142 men aged 37.00 ± 9.80 years) were those whose working schedules rotated or whose typical working hours included times before 7 a.m. or after 6 p.m. SWs were classified as regular or irregular according to the presence or absence, respectively, of rules governing work schedules (13, 16). If a consistent predetermined rule was in effect, the workers were classified as regular SWs (*n* = 2,415; 1,079 women and 1,336 men aged 37.77 ± 9.96 years). On the other hand, if there was no fixed rule or the schedules were unpredictable and inconsistent, the workers were classified as irregular SWs (*n* = 2,146; 1,340 women and 806 men aged 36.12 ± 9.64 years). To compare SWs with rotating and fixed schedules, regular SWs were classified as regularly rotating SWs, fixed evening SWs, or fixed night SWs. Regularly rotating SWs (*n* = 2,040; 886 women and 1,154 men aged 37.55 ± 9.80 years) had schedules that regularly changed over time. Fixed evening SWs (*n* = 212; 135 women and 77 men aged 38.63 ± 10.24 years) worked fixed hours (typically from 6 p.m. to 11 p.m.). Fixed night SWs (*n* = 163; 58 women and 105 men aged 39.44 ± 11.33 years) typically worked from 11 p.m. to 7 a.m. Each participant was asked to choose the category that most closely matched his/her work schedule.

### Instruments

All questionnaires were completed online. Participants could not proceed to the next question if they did not provide an appropriate answer to the current question. Information on demographic variables (e.g., age, income, gender, type of occupation, and weekly work hours) was collected from all participants. The participants were requested to indicate whether they had any medical or physical illnesses. The question about daily coffee consumption was scored as follows: 1, less than one cup; 2, one or two cups, 3: three or four cups; and 4, at least five cups. The income range was divided into two categories based on a cutoff of 3 million won, which is considered to be a middle-class income in South Korea (17). Occupations were divided into categories A (managers, professionals, and clerks), B (service and sales workers), and C (skilled agricultural forestry and fishery workers, craft and related trade workers, and elementary workers) based on the Korean Standard Classification of Occupations (KSCO) (17).

The Korean version of the Horne and d’Östberg Morningness-Eveningness Questionnaire (MEQ-K) (18) was used to measure circadian preferences. The MEQ-K has 19 items, with total scores ranging from 16 to 86. Higher scores indicate greater morningness. The Korean version of the Insomnia Severity Index (ISI) (19, 20) was also administered. The ISI comprises seven questions that assess insomnia severity, and scores range from 0 to 28; higher scores are associated with more severe insomnia. The Korean version of the Epworth Sleepiness Scale (ESS) assesses daytime sleepiness (21, 22). The eight items comprising the ESS evaluate the likelihood of falling asleep or dozing off in different situations and during various activities, including sitting and reading, watching television, and sitting in a car (as a passenger). Scores range from 0 to 24; higher scores indicate greater daytime sleepiness. The Korean version of the Pittsburgh Sleep Quality Index (PSQI) was used to measure sleep quality (23). The PSQI consists of 19 items across seven domains: subjective sleep quality, sleep latency, sleep duration, sleep efficiency, sleep disturbance, use of sleeping medication, and daytime dysfunction. Higher scores indicate worse sleep quality. The short form of the Center for Epidemiological Studies-Depression Scale (CES-D; Korean version) was used to measure depressive symptoms (24, 25). The CES-D has 11 items, with total scores ranging from 0 to 33; higher scores indicate more severe symptoms.

### Statistical analysis

Independent *t*-tests and analysis of covariance (ANCOVA; covariates = age, gender, income, type of occupation, and weekly work hours) were used to compare dimensional variables (sex and occupation) among the groups. Chi-squared tests were performed to compare categorical variables among groups. Skewness and kurtosis were evaluated when examining data normality (26). The Bonferroni correction was applied in a *post-hoc* analysis of group differences revealed using ANCOVA. Partial correlations between the MEQ and other variables were derived after controlling for age, gender, income, occupation, and weekly work hours. All analyses were performed using SPSS software (ver. 21.0; IBM Corp., Armonk, NY, United States). A *p*-value of 0.05 was taken to indicate statistical significance.

## Results

### Comparisons between non-SWs and SWs

**Figure 1:**
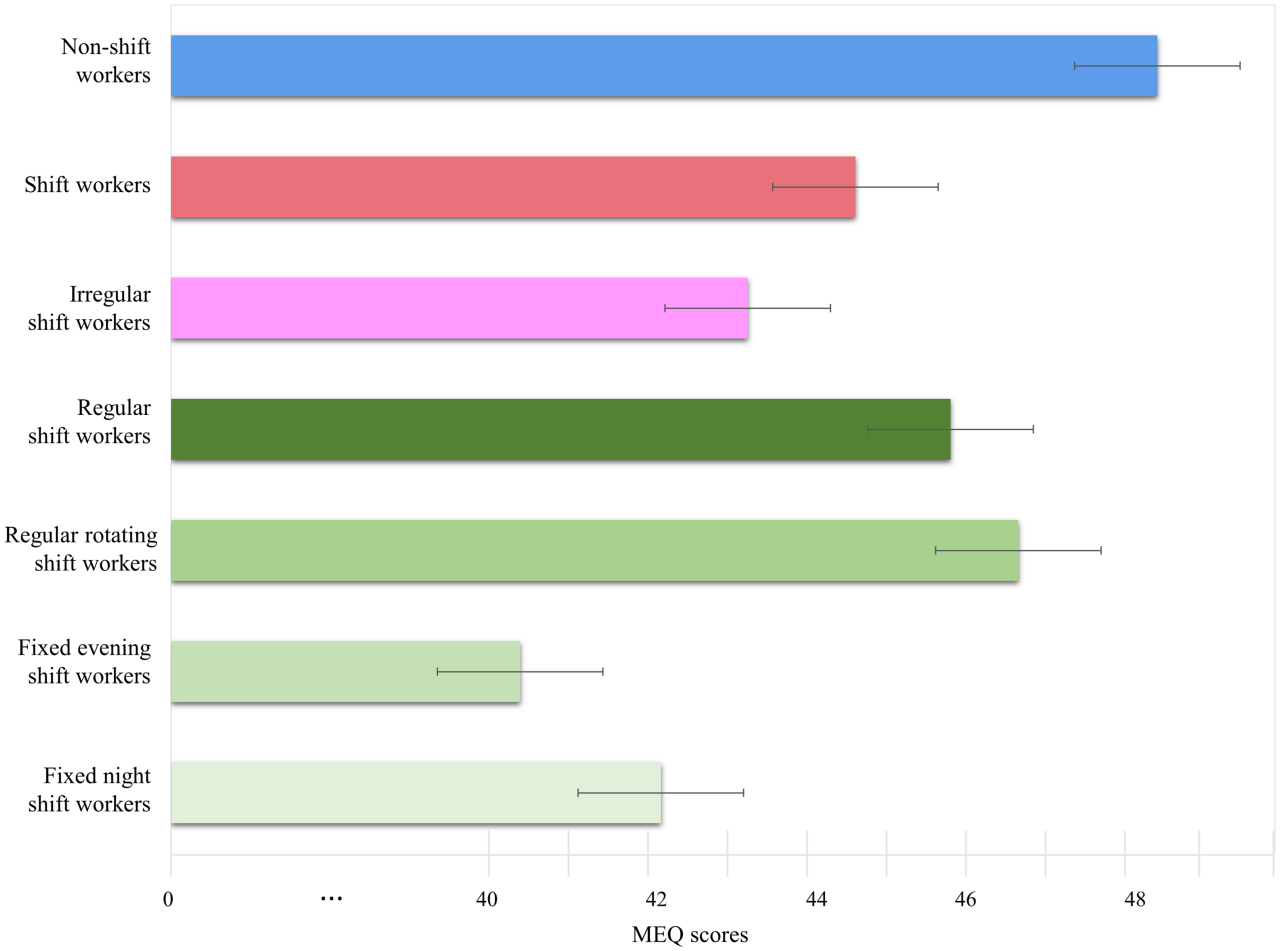
Circadian preferences by shift work type. The Morningness-Eveningness Questionnaire (MEQ) scores of shift workers (SWs) with different work schedules. The bars indicate mean MEQ scores, while the whiskers indicate standard deviations. MEQ scores of non-SWs (blue bars), SWs (red bars), irregular SWs (pink bars), regular SWs (dark green bars), regularly rotating SWs (green bars), fixed evening SWs (light green bars), and fixed night SWs (bright green bars) are shown.

SWs were significantly younger than non-SWs (t = 3.10, *p* < 0.01). Gender distribution and income did not differ between non-SWs and SWs. SWs had higher coffee consumption compared with non-SWs (*χ^2^* = 8.07, *p* < 0.05). The MEQ score was significantly higher in non-SWs than SWs after controlling for age, gender, income, occupation, and weekly work hours (*F* = 87.97, *p* < 0.001) (Table 1; Figure 1). The CES-D, ISI, and ESS scores were significantly higher in SWs than in non-SWs (CES-D, *F* = 72.59, *p* < 0.001; ISI, *F* = 129.98, *p* < 0.001; ESS, *F* = 18.45, *p* < 0.001). SWs had significantly higher PSQI scores compared with non-SWs (*t* = −13.25, *p* < 0.001).

**Table 1:** Circadian preferences, depression, and sleep disturbances among non-shift and shift workers.

	Non-shift workers (*n* = 2,093)	Shift workers (*n* = 4,561)	*F*	*p*-value
Age (years) *(mean ± SD)*	37.80 ± 9.73	36.99 ± 9.84	*t* = 3.101	0.002
Gender (male) *[n (%)]*	999 (47.7%)	2,142 (47.0%)	*χ^2^* = 0.34	0.56
Personal income [*n* (*%*)]				
*<3 million KRW/month*	1,339 (64%)	2,819 (61.8%)	*χ^2^* = 2.88	0.09
*≥3 million KRW/month*	754 (36%)	1,742 (38.2%)		
MEQ+ *(mean ± SD)*	47.15 ± 7.87	44.96 ± 8.09	87.97	<0.001
CES-D+ *(mean ± SD)*	7.11 ± 5.84	8.75 ± 6.30	72.59	<0.001
ISI+ *(mean ± SD)*	7.98 ± 5.82	10.15 ± 6.17	129.98	<0.001
ESS+ *(mean ± SD)*	7.83 ± 3.88	8.36 ± 3.97	18.45	<0.001
PSQI *(mean ± SD)*	6.25 ± 3.23	7.42 ± 3.59	121.62	<0.001

ANCOVA controlling for age, gender, income, occupation, and weekly work hours.MEQ, Morningness-Eveningness Questionnaire; CES-D, Center for Epidemiological Studies-Depression scale; ISI, Insomnia Severity Index; ESS, Epworth Sleepiness Score.

### Comparisons among non-SWs, regular SWs, and irregular SWs

Age differed significantly among the groups (*F* = 21.07, *p* < 0.01) (Table 2). Irregular SWs were significantly younger than non-SWs and regular SWs (all *p* < 0.001). Gender distribution differed significantly among the groups (*χ^2^* = 144.19, *p* < 0.001). Regular SWs were more likely to be men than non-SWs (*p* < 0.001). Non-SWs were more likely to be men than irregular SWs (*p* < 0.001). Incomes differed significantly among the groups (*F* = 11.58, *p* < 0.001). Irregular SWs earned more than non-SWs (*p* < 0.05) and regular SWs (*p* < 0.001).

**Table 2:** Circadian preferences, depression, and sleep disturbances among non-shift workers, regular shift workers, and irregular shift workers.

	Non-shift workers (*n* = 2,093)	Regular shift workers (*n* = 2,415)	Irregular shift workers (*n* = 2,146)	*F*	*p*-value	
Age (years) *(mean ± SD)*	37.80 ± 9.73	37.77 ± 9.96	36.12 ± 9.64	21.07	0.001	Non-SWs > irregular SWs^***^ Regular SWs > irregular SWs^***^
Gender (male) *[n (%)]*	999 (47.7%)	1,336 (55.3%)	806 (37.6%)	*χ*^2^ = 144.19	<0.001	Regular SWs > non-SWs > irregular SWs^***^
Personal income [*n* (*%*)]						
*<3 million/KRW month*	1,339 (64%)	1,566 (64.8%)	1,253 (58.4%)	11.58	<0.001	Irregular SWs > non-SWs ^*^ Irregular SWs > regular SWs^***^
*≥3 million KRW /month*	754 (36%)	849 (35.2%)	893 (41.6%)			
MEQ+ *(mean ± SD)*	47.15 ± 7.87	45.65 ± 7.97	44.18 ± 8.15	50.89	<0.001	Non-SWs > regular SWs^***^. Non-SWs > irregular SWs^***^. Regular SWs > irregular SWs^**^.
CES-D+ *(mean ± SD)*	7.11 ± 5.84	8.49 ± 6.28	9.03 ± 6.31	41.14	<0.001	Regular SWs > non-SWs^***^. Irregular SWs > non-SWs^***^. Irregular SWs > regular SWs^*^.
ISI+ *(mean ± SD)*	7.98 ± 5.82	9.87 ± 6.11	10.48± 6.23	73.49	<0.001	Regular SWs > non-SWs^***^. Irregular SWs > non-SWs^***^. Irregular SWs > regular SWs^**^.
ESS+ *(mean ± SD)*	7.83 ± 3.88	8.26 ± 3.97	8.48 ± 3.96	10.41	<0.001	Regular SWs > non-SWs^**^ Irregular SWs > non-SWs^***^
PSQI^+^ *(mean ± SD)*	6.25 ± 3.23	7.2 ± 3.57	7.66 ± 3.61	69.59	<0.001	Regular SWs > non-SWs^***^ Irregular SWs > non-SWs^***^ Irregular SWs > regular SWs^***^

^b^Bonferroni correction: ^*^p < 0.05; ^**^p < 0.01, ^***^p < 0.001.^+^ANCOVA controlling for age, gender, income, occupation, and weekly work hours. Bonferroni corrections were applied during *post-hoc* analyses.MEQ, Morningness-Eveningness Questionnaire; CES-D, Center for Epidemiological Studies-Depression scale; ISI, Insomnia Severity Index; ESS, Epworth Sleepiness Score, non-SWs, non-shift workers; regular SWs, regular shift workers; irregular SWs, irregular shift workers.

The MEQ scores differed significantly among the groups after controlling for age, gender, income, occupation, and weekly work hours (*F* = 50.89, *p* < 0.001). Non-SWs exhibited higher MEQ scores than regular SWs and irregular SWs (all *p* < 0.001). Regular SWs had higher MEQ scores than irregular SWs (*p* < 0.01). The ISI scores differed significantly among the groups after controlling for age, gender, income, occupation, and weekly work hours (*F* = 73.49, *p* < 0.001). Irregular SWs had higher ISI scores than regular SWs and non-SWs (all *p* < 0.01). The ESS scores differed significantly among the groups after controlling for age, gender, income, type of occupation, and weekly work hours (*F* = 10.41, *p* < 0.001). Regular and irregular SWs had higher ESS scores than non-SWs (*p* < 0.01 and *p* < 0.001, respectively). The PSQI scores differed significantly among the groups after controlling for age, gender, income, types of occupation, and weekly work hours (*F* = 76.03, *p* < 0.001). Irregular SWs exhibited higher PSQI scores compared with non-SWs and regular SWs (all *p* < 0.001). Regular SWs had higher PSQI scores compared with non-SWs (*p* < 0.001). The CES-D scores differed significantly among the groups after controlling for age, gender, income, occupation, and weekly work hours (*F* = 41.14, *p* < 0.001). Regular and irregular SWs had higher CES-D scores than non-SWs (all *p* < 0.001). Irregular SWs had higher CES-D scores than regular SWs (*p* < 0.05) (Table 3).

**Table 3:** Circadian preferences, depression, and sleep disturbance scores of fixed evening shift workers, fixed night shift workers, and regularly rotating shift workers.

	Regularly rotating shift workers (*n* = 2,040)	Fixed evening shift workers (*n* = 212)	Fixed night shift workers (*n* = 163)	*F*	*p*-value	
Age (years) *(Mean ± SD)*	37.55 ± 9.80	38.63 ± 10.24	39.44 ± 11.33	6.57	0.03	
Gender (men) *[n (%)]*	1,154 (56.6%)	77 (36.3%)	105 (64.4%)	*χ^2^* = 14.67	<0.001	Regularly rotating SWs > fixed evening SWs^***^; Fixed night SWs > fixed evening SWs^***^
Personal Income [*n* (*%*)]						Regularly rotating SWs > fixed evening SWs^**^; Regularly rotating SWs > fixed night SWs^**^
*<3 million/month (KRW)*	1,278 (62.6%)	163 (76.9%)	125 (76.7%)	24.08	<0.001	
*≥3 million/month (KRW)*	762 (37.4%)	49 (23.1%)	38 (23.3%)			
MEQ+ *(mean ± SD)*	46.14 ± 7.72	42.53 ± 8.82	43.55 ± 8.69	22.42	<0.001	Regularly rotating SWs > fixed evening SWs^***^; Regularly rotating SWs > fixed night SWs^***^
CES-D+ *(mean ± SD)*	8.36 ± 6.23	8.76 ± 6.35	9.84 ± 6.69	3.79	0.01	Fixed night SWs > regularly rotating SWs^*^
ISI+ *(mean ± SD)*	9.82 ± 6.02	9.53 ± 6.39	10.88 ± 6.71	4.00	0.007	–
ESS+ *(mean ± SD)*	8.33 ± 3.98	7.84 ± 4.06	8.01 ± 3.78	0.83	0.48	–
PSQI^+^ *(mean ± SD)*	7.18 ± 3.53	6.97 ± 3.67	7.72 ± 3.87	2.36	0.07	

^b^Bonferroni correction, ^*^p < 0.05, ^**^p < 0.01, ^***^p < 0.001.^+^ANCOVA controlling for age, gender, income, occupation, and weekly hours worked.Bonferroni corrections were applied during *post-hoc* analyses. MEQ, Morningness-Eveningness Questionnaire; CES-D, Center for Epidemiological Studies-Depression scale; ISI, Insomnia Severity Index; ESS, Epworth Sleepiness Score; regularly rotating SWs, regularly rotating shift workers; fixed evening SWs, evening shift workers; fixed night SWs, night shift workers.

### Comparisons among regular SWs

Age did not differ between fixed night and regularly rotating SWs. Gender distribution differed significantly among the groups (*χ2* = 14.67, *p* < 0.001). Fixed night SWs were more likely to be men (64.4%) than regularly rotating SWs and fixed evening SWs (all *p* < 0.001). Regularly rotating SWs were more likely to be men than fixed evening SWs (*p* < 0.01). Incomes differed significantly among the groups (*F* = 24.08, *p* < 0.001). Regularly rotating SWs earned more than fixed evening SWs (*p* < 0.01) and fixed night SWs (*p* < 0.01).

The MEQ scores differed among regularly rotating SWs, fixed evening SWs, and fixed night SWs after controlling for age, gender, income, occupation, and weekly work hours (*F* = 22.42, *p* < 0.001). Regularly rotating SWs had higher MEQ scores than fixed evening and fixed night SWs (all *p* < 0.001). There was no significant difference in MEQ scores between fixed evening and fixed night SWs. The CES-D scores differed among regularly rotating SWs, fixed evening SWs, and fixed night SWs after controlling for gender, income, occupation, and weekly work hours (*F* = 3.79, *p* < 0.05). Fixed night SWs had higher CES-D scores than regularly rotating SWs (*p* < 0.05). The ISI, ESS, and PSQI scores did not differ among regularly rotating SWs, fixed evening SWs, and fixed night SWs after controlling for gender, income, occupation, and weekly work hours.

### Relationships of circadian preference with depression and sleep

**Figure 2:**
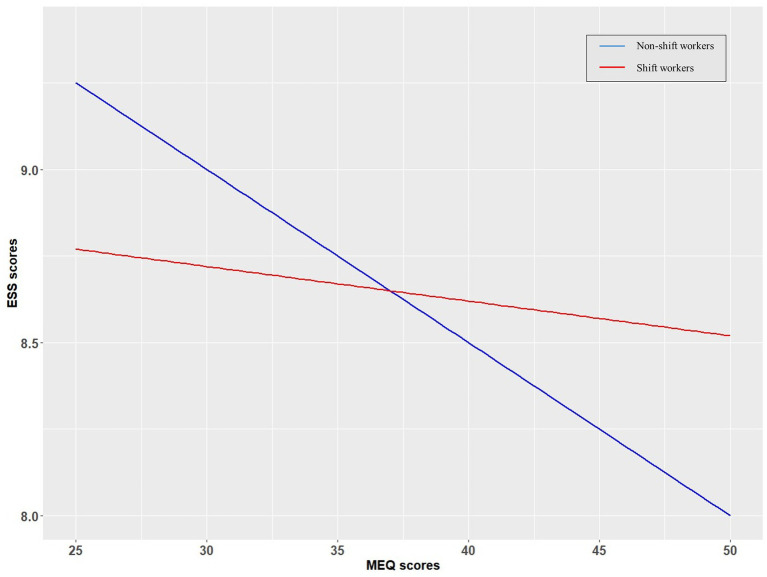
Relationships between circadian preferences and daytime sleepiness in shift workers (SWs) and non-SWs. Linear trendlines of SWs and non-SWs. X-axis: MEQ scores, y-axis: ESS scores. Red line: linear trendline of SWs. Blue line: linear trendline of non-SWs. MEQ, Morningness-Eveningness Questionnaire; ESS, Epworth Sleepiness Scale.

The MEQ scores were significantly associated with the CES-D scores in all types of SWs (all participants, r = −0.13, *p* < 0.001; non-SWs, r = −0.13, *p* < 0.001; all SWs, r = −0.12, *p* < 0.001; irregular SWs, r = −0.09, *p* < 0.001; all regular SWs, r = −0.13, *p* < 0.001; regularly rotating SWs, r = −0.12, *p* < 0.001; fixed evening SWs, r = −0.18, *p* < 0.05; fixed night SWs, r = −0.17, *p* = 0.03) (Table 4). The MEQ scores were significantly associated with the ISI scores in all types of SWs (all participants, r = −0.20, *p* < 0.001; non-SWs, r = −0.16, *p* < 0.001; all SWs, r = −0.20, *p* < 0.001; irregular SWs, r = −0.17, *p* < 0.001; all regular SWs, r = −0.22, *p* < 0.001; regularly rotating SWs, r = −0.20, *p* < 0.001; fixed evening SWs, r = −0.25, *p* < 0.001; fixed night SWs, r = −0.27, *p* < 0.01) (Table 4). The PSQI scores were significantly associated with the MEQ scores (all participants, r = −0.21, *p* < 0.001; non-SWs, r = −0.21, *p* < 0.001; all SWs, r = −0.20, *p* < 0.001; irregular SWs, r = −0.19, *p* < 0.001; all regular SWs, r = −0.21, *p* < 0.001; regular rotating SWs, r = −0.20, *p* < 0.001; fixed evening SWs, r = −0.29, *p* < 0.001; fixed night SWs, r = −0.20, *p* < 0.05) after controlling for age, gender, income, occupation, and weekly work hours. The ESS and MEQ scores were significantly associated in non-SWs (r = −0.85, *p* < 0.0001) but not in any type of SW (Table 4; Figure 2). Coffee consumption was significantly negatively associated with the MEQ scores after controlling for age, gender, income, occupation, and weekly work hours in non-SWs (r = −0.08, *p* < 0.001), SWs (r = −0.04, *p* < 0.001), regular SWs (r = −0.05, *p* < 0.001), regular rotating SWs (r = −0.05, *p* < 0.01), and fixed evening SWs (r = −0.12, *p* < 0.05). The ESS scores were correlated with the MEQ scores in non-SWs (r = −0.09, *p* < 0.001) but not in any type of SW, after controlling for age, gender, income, occupation, weekly work hours, illness, and coffee consumption. The ESS scores were correlated with the MEQ scores in non-SWs (r = −0.05, *p* < 0.05) and regular rotating SWs (r = −0.05, *p* < 0.05), but not in irregular SWs, fixed evening SWs, or fixed night SWs, after additionally controlling for PSQI scores. The ESS and MEQ scores were significantly correlated with each other after additionally controlling for ISI scores in non-SWs (r = −0.57, *p* < 0.01) and regular rotating SWs (r = 0.06, *p* < 0.01) but not in irregular SWs, fixed evening SWs, or fixed night SWs.

**Table 4:** Relationships among circadian preference, depression, and sleep disturbance scores according to shift work type.

	All subjects	Non-shift workers	Shift workers					
			All shift workers	Irregular shift workers	Regular shift workers			
					All regular shift workers	Regularly rotating shift workers	Fixed evening shift workers	Fixed night shift workers
CES-D	r = −0.13 *p* < 0.001	r = −0.13 *p* < 0.001	r = −0.12 *p* < 0.001	r = −0.09 *p* < 0.001	r = −0.13 *p* < 0.001	r = −0.12 *p* < 0.001	r = −0.18 *p* = 0.01	r = −0.17 *p* = 0.03
ISI	r = −0.20 *p* < 0.001	r = −0.16 *p* < 0.001	r = −0.20 *p* < 0.001	r = −0.17 *p* < 0.001	r = −0.22 *p* < 0.001	r = −0.20 *p* < 0.001	r = −0.25 *p* < 0.001	r = −0.27 *p* = 0.001
ESS	r = −0.31 *p* = 0.11	r = −0.85 *p* < 0.001	r = 0.00 *p* = 0.92	r = −0.01 *p* = 0.70	r = 0.01 *p* = 0.50	r = 0.00 *p* = 0.99	r = 0.02 *p* = 0.75	r = 0.05 *p* = 0.56
PSQI	r = − 0.21 *p* < 0.001	r = − 0.21 *p* < 0.001	r = − 0.20 *p* < 0.001	r = −0.19 *p* < 0.001	r = −0.21 *p* < 0.001	r = −0.20 *p* < 0.001	r = −0.29 *p* < 0.001	r = −0.20 *p* < 0.05

r: correlation coefficients; ^*^p < 0.05, ^**^p < 0.01, ^***^p < 0.001.Partial correlation analyses controlled for age, gender, income, occupation, and weekly hours worked.MEQ, Morningness-Eveningness Questionnaire; CES-D, Center for Epidemiological Studies-Depression scale; ISI, Insomnia Severity Index; ESS, Epworth Sleepiness Score.

## Discussion

We found that SWs exhibited more eveningness than non-SWs, particularly those with irregular or fixed evening/night shifts. An association between eveningness and daytime sleepiness was apparent in non-SWs but not in SWs. The greater eveningness of SWs is consistent with our first hypothesis. There are two possible explanations for this result. First, eveningness SWs may be more likely to choose night shift work than morningness SWs. Previous studies demonstrated that morningness individuals found it difficult to adjust to shift work and were less likely than eveningness individuals to engage in such work (27). People with morningness exhibited slower circadian adjustment to night work than those with eveningness (28); moreover, they experienced more sleep deprivation and greater fatigue (29) and were sleepier during night work (9). Taken together, such findings suggest that eveningness individuals choose shift work more often than morningness individuals because they experience less difficulty adapting to it.

Another possibility is that the circadian preferences of SWs may change after beginning shift work. As shift work differs from work during normal hours, circadian rhythm readjustment may be required. Repeated readjustments necessitated by shift work may change the circadian preference. During adaptation to night work, sleepiness during work decreased, and bedtime was gradually delayed (30). A previous study measured the so-called “melatonin metabolite acrophase,” which corresponds to the maximum melatonin concentration during a 24 h period (31). In that study, the melatonin metabolite acrophase changed after seven night shifts, and the change persisted even after SWs returned to day shifts. This result suggests that repeated night shifts may induce prolonged changes in the circadian rhythm and, potentially, the circadian preference. However, we cannot confirm a change in circadian preference after repeated shift work due to a lack of long-term follow-up data for this study.

We found that irregular SWs exhibited more eveningness than regular SWs. A previous study reported that irregular SW delayed the sleep midpoint (32), i.e., the circadian rhythm. A delayed circadian rhythm is closely associated with eveningness. As eveningness individuals exhibit more flexible sleep–wake cycles (33), eveningness SWs may experience less difficulty than others in adapting to irregular work schedules and may thus be more willing to choose such schedules. It is also possible that irregular schedules may increase the eveningness tendency. In previous studies, delayed sleep midpoints were observed when daily routines were disturbed (34), and irregular sleep and light exposure were associated with delayed circadian rhythms in college students (35). Moreover, the erratic irregular schedules of SWs may disrupt the circadian rhythm and sleep–wake cycle (36). However, it remains unclear whether irregular SW delays the circadian rhythm or induces eveningness.

We compared the eveningness of fixed evening and night SWs to that of regularly rotating SWs. The work schedules of the latter SWs usually included both day and night shifts; fixed night or evening SWs worked only at night or in the evening. Our findings suggest that circadian preference may modulate the tolerability of fixed night or rotating schedules. A previous study reported that fixed night work was more acceptable than rotating night work (37). However, among regular SWs, those with morningness may be more tolerant of rotating schedules, whereas those with eveningness may be more accepting of fixed night schedules. Eveningness is associated with greater night alertness and morning sleepiness; it is thus not surprising that eveningness individuals are more likely to choose fixed night/evening shifts than rotating day–night shifts. Another possible explanation for the higher eveningness among fixed evening/night SWs is that evening/night SWs might change their circadian preferences. A previous longitudinal study found that the number of night shifts worked over 2 years correlated with increased eveningness over time (38). Thus, repeated evening shifts may lead to a circadian preference for eveningness.

We found that non-SWs exhibiting evening preferences experienced higher levels of daytime sleepiness than non-SWs with morning preferences. However, we observed no correlation between SW circadian preference and sleepiness. In previous studies, eveningness subjects reported more daytime sleepiness than morningness individuals (9). Thus, the correlation between eveningness and daytime sleepiness in non-SWs is not surprising. However, we found no correlation between eveningness and daytime sleepiness among SWs. It may be that eveningness SWs are sleepiness-resistant and thus better handle varying shift schedules. Night shift sleepiness was less severe in eveningness than morningness individuals in a previous study (39). Moreover, a reduction in daytime sleepiness reflects the extent of adaptation (40); our findings thus support those of previous studies reporting that eveningness was associated with easier adaptation to shift work (9, 41). The less severe shift work-induced sleepiness in eveningness subjects may reduce the association between eveningness and sleepiness often seen in the general population.

This study had some limitations. First, cross-sectional studies cannot identify temporal or causal associations between SW schedules and circadian preferences. In other words, we cannot state that individuals exhibiting eveningness tended to choose specific types of shift work or that shift work changed their circadian preferences; longitudinal studies are required. Second, all data were self-reported and may have been affected by selection bias because all subjects volunteered to take part. Future studies using objective measures, such as structured interviews or polysomnography, would help validate our results. Additionally, studies regarding the correlations of biological circadian rhythm indices (e.g., melatonin, cortisol, and core body temperature) with circadian preferences in SWs could provide insights into those preferences. Third, workers with rotating shift schedules limited to daytime hours (7 a.m.–6 p.m.) were classified as SWs in this study, although such workers in Korea are rare.

## Conclusion

SWs, especially those on irregular/nighttime shifts, exhibited more eveningness than non-SWs. A correlation between circadian preference and daytime sleepiness was evident in non-SWs but not SWs. Circadian preference was associated with shift work and the schedules thereof, but not with SW sleepiness.

## Data availability statement

The original contributions presented in the study are included in the article/supplementary material, further inquiries can be directed to the corresponding author.

## Ethics statement

The studies involving humans were approved by Institutional Review Board of Samsung Medical Center (approval no. 2019-04-095). The studies were conducted in accordance with the local legislation and institutional requirements. The participants provided their written informed consent to participate in this study. Written informed consent was obtained from the individual(s) for the publication of any potentially identifiable images or data included in this article.

## Author contributions

JA: Conceptualization, Data curation, Formal analysis, Visualization, Writing – original draft. HY: Writing – review & editing, Data curation, Validation, Formal analysis. SL: Data curation, Writing – review & editing, Methodology, Formal analysis. YH: Data curation, Investigation, Writing – review & editing, Methodology. SJ: Data curation, Writing – review & editing, Methodology, Conceptualization. SK: Writing – original draft, Writing – review & editing, Conceptualization, Data curation, Funding acquisition, Investigation, Project administration, Resources, Supervision, Validation.
